# Experiencing Violence among Children and Adolescents with Depression in the Aspect of Polish Law

**DOI:** 10.3390/jcm11195818

**Published:** 2022-09-30

**Authors:** Aleksandra Lewandowska, Katarzyna Bliźniewska-Kowalska, Piotr Gałecki, Rafał Kubiak

**Affiliations:** 1 Child Psychiatry Ward, Babinski Hospital in Lodz, 91-229 Lodz, Poland; 2 Department of Adult Psychiatry, Medical University of Lodz, 90-419 Lodz, Poland; 3 Department of Criminal Law, University of Lodz, 90-033 Lodz, Poland

**Keywords:** bullying, depression, child, adolescents, denunciation, medical secret

## Abstract

Violence is not uncommon in the contemporary world. The consequences of harmful experiences in childhood are often educational problems, difficult behavior, failure to cope in adulthood, duplication of learned, negative behavior patterns and disorders in various spheres/areas of life. The experience of childhood violence is associated with the occurrence of about half of mental disorders with onset in childhood and one third of disorders that appear later in life. Various emotional and behavioral disorders are mentioned among the psychological effects of violence against a child, including depressive disorders. Regarding experiences of violence, there is strong evidence that exposure to sexual or physical violence is a predictor of depressive episodes and depressive symptoms in adolescents. Among adolescents, the impact of violence on depression has been shown to be sustained. Accordingly, evidence suggests that elevated depressive symptoms and episodes of depression may even persist for up to two years after experiencing cases of violence. Due to the destructive consequences of such behavior, international and national law devote much attention to the protection of children’s rights. Under Polish law, there are regulations describing measures of reaction within the family, as well as provisions sanctioning violent behavior. Therefore, the study discusses the family and criminal law aspects of violence against minors. The whole study is imbued with considerations of the so-called the obligation to denounce, i.e., to notify about the disclosure of a prohibited act committed to the detriment of minors. This issue was presented in the context of medical secrets and its type—psychiatric discretion.

## 1. Introduction

In today’s world, violence is not uncommon. It is not only national, global or universal, but also historical, because its origins would be difficult to find in contemporary phenomena.

Violence against children is quite a common and disturbing phenomenon also occurring in Poland. Adults, not coping with their problems, look for opportunities to react to their emotions against children who become victims of their mental, moral and social immaturity. The consequences of harmful experiences in childhood often include educational problems, difficult behavior, an inability to cope with problems in adult life, duplicating learned, negative behavior patterns and disorders in various areas of life [1]. The World Health Organization states that child abuse is any intentional or unintentional act of an adult or community that has a detrimental effect on the health, physical or psychosocial development of a child, and that violence is the deliberate use of physical force or power, formulated as a threat or actually used, directed against oneself, another person, group or community, which either leads to or is associated with a high probability of causing bodily injury, death, psychological damage, developmental defects or lack of elements necessary for normal life and health [2].

The DSM-5 classifies violence and neglect under “Other Conditions That May Be of Clinical Interest”. Within this chapter, there is a section titled Problems of Abuse and Neglect, including physical abuse of a child, sexual abuse of a child, neglect of a child, and psychological abuse of a child [3]. In Poland, statistics on violence include information on committed and reported abuse of children, while there are still unreported experiences and those about which minors have not told anyone [4]. According to statistics, mental and physical violence is the most frequently reported during an intervention. Boys are more likely to experience physical and girls more psychological abuse. Parents are most often the perpetrators of physical and mental abuse [4].

The aim of the study is to draw attention to the relationship between the experience of violence and symptoms of depression in childhood, adolescence and adulthood, based on the reports of scientists and clinicians in relation to their research in this area. The developed conclusions draw attention to a quite significant social problem and the far-reaching effects of the use of violence against children and the need to take preventive and therapeutic measures, including the protection of children’s rights. In view of the above, this article also notes that due to the destructive consequences of the use of violence against minors, international and national law devotes a lot of attention to this issue, which is important in the area of the preventive measures taken.

## 2. Types of Violence

Physical violence is aggressive behavior that violates the child’s physical integrity. Abuses take various forms, from the more delicate type of slap, jerking, to more severe beating, kicking and even torture [5]. Sexual violence against children is any sexual behavior aimed at satisfying the sexual needs of an adult. Abuses are divided into violence without physical contact, such as peeping, watching or showing private parts of the body, and talking about sexual topics. The other is sexual arousal by touching the child or forcing the child to touch the abuser. The next are various types of sexual relations and the exploitation of children for pornographic purposes, prostitution and the abuse of various forms of violence. We also divide sexual violence into two types. The first is domestic violence where the perpetrator is a family member or legal guardian. The second type is intra-family violence, when the abuser is a person known to the child or not [5]. Psychological violence is a type that is difficult to define and depends on social norms, culture or upbringing. This type of harm is described as deliberately undermining a child’s development. The use of emotional violence may be conscious behavior towards a minor or unconscious harm that compensates an adult for his needs. Emotional abuse is defined as humiliating, ridiculing, scaring, ignoring, name-calling and other forms of hostile and rejecting treatment [5]. Child neglect is defined as a deliberate or unintentional effect and is associated with a threat to the conditions of proper mental, physical and social development. It consists of the failure to create appropriate developmental conditions for the child, as well as ignoring emotional, health, educational, nutritional, or safety needs [5].

The experience of violence disrupts the proper emotional development of a child, negatively affecting information processing about emotional color, leading to specific disorders in the recognition, understanding and expression of emotions, and sometimes to deficits in empathy and pro-social behavior. Violence and has a negative impact on the psychological functioning of children, causing disturbances in relationships, personality development and emotional regulation, as well as maladaptive coping and risky behavior. The experience of childhood violence is associated with the occurrence of about half of mental disorders with onset in childhood and one third of disorders that appear later in life. The psychological effects of violence against a child include various emotional and behavioral disorders, such as anxiety disorders, depressive disorders, sleep and appetite disorders, psychosomatic disorders, eating disorders, enuresis, self-harm and suicidal behavior, addictions, aggressive behavior and relationship, social and personality disorders. Moreover, it is associated with a higher incidence of chronic diseases and a higher risk of premature death [6]. Although emotional abuse is not as visible as other forms of violence, it is considered a significant threat to the mental and physical health of the victim. A child growing up in a harmful and neglectful environment perceives the world as threatening and unstable and experiences a sense of helplessness and hopelessness. As a result, children may fall into states of numbness, withdrawal, or over-excitement. Criticism, humiliation, and neglecting basic emotional needs contribute to the development of lowered self-esteem and a sense of worthlessness. Victims of emotional abuse are at risk of developing various social and emotional problems as well as personality disorders. They are at an increased risk of both developing mental disorders, e.g., depression, anxiety disorders, eating disorders, and adopting violent and aggressive behaviors, and alcohol or other psychoactive substance abuse. They have problems with coping with stress, anxiety and impaired social skills development. In adulthood, people who have been victims of emotional abuse in childhood have a diminished ability to establish stable and supportive relationships with their children and are more likely to become abusers. Victims of emotional abuse often have problems in their educational functioning, which are manifested by lower learning outcomes or difficulties in the cognitive sphere (remembering, IQ) [6].

## 3. Depression among Children and Adolescents—The Scale of the Phenomenon

Depression is the leading cause of disability worldwide and is estimated to be a major contributor to the global burden of disease by 2030. [7] The negative consequences of depression are not limited to mental stress as it is associated with serious health problems and it also increases the risk of death by suicide [8]. The widespread impact of depression may be partly due to its particularly high incidence in adolescence, which causes relentless and cumulative suffering throughout life [9], and adolescents are undoubtedly particularly vulnerable to depression [10]. Epidemiological data indicate that as many as 1 in 5 adolescents will experience depressive disorders [11], and there is evidence that rates of depression in adolescence are increasing [12,13]. The severity of depressive symptoms in adolescence disproportionately affects girls at the ages of 13–15 years, with prevalence rates doubling in girls aged 15–18 [14,15]. In addition to the increased incidence of depression in racially and ethnically diverse low-income adolescent girls, the risk of depression increases significantly with childhood abuse [16,17,18]. Although the prevalence of depression in the United States is estimated to be 7%, the rates of depression among those with a history of childhood abuse are much higher [19]. Increased rates of depressive symptoms were also associated with families with lower income, as well as in underrepresented minority groups [20].

People with a history of abuse may experience difficulties in relationships with parents, peers, teachers and romantic partners [20,21,22]. In turn, these relational difficulties may increase the risk of depression developing during adolescence, the developmental period when relationships outside the home become particularly important. Research has also shown that children who have experienced multiple subtypes of abuse (e.g., physical abuse, neglect, sexual abuse, etc.) are even more likely to develop depressive symptoms [18]. Moreover, adolescents with a history of trauma have been shown to be more stress-responsive to life events that are less severe than teens who do not have a history of abuse [23]. Serious sexual abuse is also associated with dysregulation of stress response systems and future responsiveness to lower stress levels [24,25,26]. Some studies have shown blunted or asymmetric physiological responses in girls who were sexually abused, who showed an increase in depressive symptoms [26]. It is therefore clear that a history of mistreatment triggers a negative cascade that adversely affects human relationships, contributes to the appearance of depressive symptoms and increases the likelihood of major depressive episodes throughout life. Moreover, it is clear that sexual abuse can be a particularly strong risk factor. Child abuse has been documented as a strong depressant risk factor [27]. Abused children often function in hostile conditions where it is known that this pathogenic environment causes various problems with adaptation, which is likely to be responsible for a strong association with depression, including difficulty in solving developmental tasks relevant at the appropriate stage, insecure attachments, difficulties with the recognition and regulation of emotions, negative emotional patterns and interpersonal challenges [27,28]. One meta-analytical review suggests that more than half of global depression can be credibly attributed to child abuse [29]. Moreover, child abuse is associated with greater chronicity, severity and duration of depression [30]. While it has been established that child abuse increases depression, additional research is needed to understand how the adverse effects of child abuse may vary under certain conditions or contexts [31]. Child abuse can create a depressive state of sensitivity that can permeate throughout the course of life and become amplified or magnified in the face of more proximal stressors [27,32,33]. Suffering from experiencing depression early in life often seriously affects later development, as evidenced, for example, by dropping out of school and lower life satisfaction [34,35]. Apart from genetic and other factors (e.g., cognitive), it is known that psychosocial stressors play an important role in the etiology of the disorder [36,37]. Regarding experiences of violence, there is solid evidence that exposure to sexual or physical violence is a predictor of depression and depressive symptoms in adolescents [38,39,40]. The impact of violence on depression among adolescents has been shown to be sustained. Consistently, evidence suggests that elevated depressive symptoms and episodes of depression may even persist for up to two years after experiencing cases of violence [40,41,42].

## 4. Experiencing Violence and Symptoms of Depression in Childhood, Adolescence and Adulthood in Light of Empirical Research

The LONGSCAN study showed that witnessing domestic violence at the age of 4, 6 and 8 was associated with depression and anxiety [42]. Furthermore, at 12 years of age, mental abuse was associated with more negative effects than other exposures; and at the age of 18, sexual abuse was the strongest predictor of negative outcomes, suggesting that different exposures have different consequences throughout the child’s life.

Russell et al. conducted a study on witnessing and the experience of childhood abuse and depression in adults [43]. The study was conducted in Miami, Florida, from 1998 to 2000 when participants were 19 to 21 years old, and from 2000 to 2002 where the majority of people were 21 to 23 years of age. A total of 1175 people participated in the study. The first part included participants’ own report on the experience; in the second part, they assessed the severity of depressive symptoms 2 years later. Witnessing violence, experiencing violence, and family structure and exposure to other adverse factors were investigated through the interview. Depressive symptoms were measured using the modified CES-D depression scale. The results of the study indicate a relationship between violence and symptoms of depression; frequent exposure to domestic violence has a significantly greater impact on the occurrence of depressive symptoms [43]. Piechaczek et al. conducted a study in a group of 100 individuals diagnosed with an episode of depression and a control group of 101 subjects matched according to gender and age. [44]. The study group was recruited from two children and adolescent psychiatry departments. According to the ICD-10 [45], 18 people had a mild depressive episode, 26 a moderate depressive episode and 56 a severe depressive episode. Patients with current or a history of attention deficit hyperactivity disorder (ADHD), schizophrenia, bipolar disorder, or other developmental disorder were excluded. Patients diagnosed with a depressive episode with other comorbid diagnoses than those mentioned above were included if depression was the primary diagnosis. The diagnosis of depression and potential comorbid mental disorders based on ICD-10 [45] was made using a standardized semi-structured interview (Diagnostisches Interview bei psychischen Störungen im Kindes-und Jugendalter Kinder-DIPS;) [46].

To assess the severity of the depressive episode, children aged 10 to 12 years completed the Depressions-Inventar für Kinder und Jugendliche DIKJ; German version [47], while adolescents over 12 years of age completed the Beck Depression Inventory-second edition (BDI-II; German version) [48]. Subjects diagnosed with depression obtained higher scores in DIKJ / BDI-II compared to the control group. A comprehensive questionnaire on psychosocial stressors was adapted from the Life Event Survey and the Munich Event List [49]. The self-assessment questionnaire assessed psychosocial stressors related to changes at home or school, death of a loved one, experiences of violence and criminal behavior. To assess protective factors, participants were given two questionnaires on social support and family climate. The social support questionnaire was adapted from the MOS Social Support Survey [50]. The questionnaire conducted to assess family relationships was taken from the children’s health survey in Germany and was based on the family climate scale (Der Kinder- und Jugendgesundheitssurvey KiGGS) [51,52]. Based on the results of studies corresponding to the reports of other researchers [38,39,40], experiences of violence, especially being beaten at home, being insulted at home and being a victim of violence, were more frequent in adolescents diagnosed with depression compared to those in the control group. It is noted that the experience of violence, especially early in life, may lead to neurobiological changes, e.g., changes reflected in the dysregulation of the hypothalamic-pituitary-adrenal (HPA) axis. This may predispose individuals to psychopathology, including increased susceptibility to the occurrence and maintenance of an episode of depression [53].

Blum et al., as part of the Global Early Adolescent Study, developed the ACE 11-point Adverse Childhood Experiences scale piloted among 1284 adolescents aged 10–14 years in low-income communities in 14 cities around the world [54]. This study shows a high exposure to difficult random events, with 45.79% reporting victimization of violence, 38.08% experiencing emotional neglect, and 29.28% reporting physical neglect. These rates are more than 10% higher than the national average for adults in the US [55]. This study also found that two extended ACEs, victimization of violence (which includes bullying) and household instability, act as exposures to ACEs and may be important to include in future ACE research during adolescence. Contrary to popular belief, boys appear to consistently report greater exposure to ACE and greater fear of physical abuse and neglect than girls. According to the literature, girls have been noticed to show greater internalizing behaviors such as depression and anxiety disorders [56,57], while boys show greater externalizing behaviors such as poor behavioral regulation and aggression [58,59]. This study shows that as in high-income countries, those exposed to more ACE as children living in the poorest parts of cities significantly increased their risk of depression and violence in their teens compared with low-exposure peers, regardless of gender. Researchers found that there was an association between cumulative ACE exposure as measured by the ACE index and negative outcomes (depressive symptoms and violence). Moreover, they found that, compared to exposure to domestic violence and instability, high exposure to neglect and physical and emotional abuse were associated with an increased likelihood of developing depressive symptoms. Researchers indicate that high exposure to neglect, in addition to physical and emotional abuse, is associated with a greater likelihood of developing depressive symptoms than exposure to victimization of violence and household instability. This finding is in line with the study by Spinazzoli et al. [60], which showed that mental abuse (including emotional neglect) was associated with an increased risk of developing depression in a national sample of adolescents. The relationship between mental maltreatment and symptoms of internalization was also supported by other studies [61,62,63]. These findings are of particular importance in developing programs for young adolescents to reduce the incidence of violence and/or depression.

Cathy Spatz Widom, Kimberly Daumont and Sally J. Czaja (2007) conducted a longitudinal study to establish the relationship between sexual and physical abuse and neglect in childhood and depression in adult life [64]. The study was conducted with a group of 1196 people. The first study was conducted when people were under 12 years of age, and the second when they were about 28 years old in the Midwestern United States; selected individuals who had a documented history of violence in court records were included. A structured interview was used to examine the disorders according to the DSM-III-R criteria. The results show a relationship between depression in adulthood and the experience of childhood abuse. People who experience physical abuse or neglect and a combination of violence have more frequent depressive states than those who do not have a history of childhood abuse.

The described studies show a relationship between the experience of violence and the occurrence of depression or depressive symptoms in childhood, adolescence and in adulthood. Attention is drawn to what is quite a significant social problem and the far-reaching effects of the use of violence against children. The identification of significant and frequent stressors in the context of depression among minors is very important as these factors can constitute specific targets in preventive and therapeutic activities.

## 5. Legal Aspects of Counteracting Violence against Children and Youth

### 5.1. Protection of Children’s Rights in Terms of International and National Law

The destructive impact that violence against minors may have, prompted legislators to undertake appropriate work aimed at introducing regulations, on the one hand, protecting children, and, on the other hand, sanctioning the perpetrators of such violence. This issue was recognized at the international level already at the beginning of the 20th century, with the acknowledgement that humanity should give the child the best it has. In particular, the Declaration ensures the provision of “normal physical and spiritual development” for the child, as well of food for children, and appropriate care in case of illness [65].

Similar values were at the heart of the International Covenant on Civil and Political Rights, opened for signature in New York on 19 December 1966 [66]. According to its Art. 24 sec. 1 “every child, without any discrimination on the basis of race, color, sex, language, religion, national or social origin, financial situation or birth, has the right to the protection measures required by the status of a minor, by family, society and the State.” The protection of the child is also guaranteed in Art. 10 sec. 3 of the International Covenant on Economic, Social and Cultural Rights, opened for signature in New York on 19 December 1966 [67]. Under this provision, States party to the Covenant were required to take special measures to ensure “protection and assistance to all children and adolescents without any discrimination on the basis of origin or other grounds”. The rights of the child are most emphatically articulated in the Convention on the Rights of the Child adopted by the United Nations General Assembly on 20 November 1989 [68]. In particular, through Art. 19, states party to the Convention are obliged to take all appropriate legislative, administrative, social and educational measures to protect the child from all forms of physical or mental violence, harm or neglect, or mistreatment or exploitation, including sexual exploitation, of the care of the parent (s), legal guardian (s) or other person caring for the child. In turn, paragraph 2 requires that all “preventive measures are taken to establish, inform, initiate and conduct investigations, proceedings, record the above-mentioned cases of child abuse and, where appropriate, court interference”. Such an influx of international legislation prompted the representatives of the doctrine to formulate the opinion that in the 20th century the protection of children’s rights became the most important idea. “Children are seen as vulnerable subjects due to their physical and mental immaturity. That is why the protection of their rights has become one of the leading aspects of human rights protection in domestic legislation (especially in democratic states and in international law” [69].

These views were also the basis of Polish law, in which the child’s welfare was even raised to the constitutional level. According to Art. 72 sec. 1 of the Constitution of the Republic of Poland, the Republic of Poland ensures protection of children’s rights. Everyone has the right to demand that the organs of public authority protect the child against violence, cruelty, exploitation and demoralization. In addition, to guard the protection of minors, the Constitution of the Republic of Poland established a special body, which is the Ombudsman for Children (its tasks and competences are described in detail in the Act of 6 January 2000 on the Ombudsman for Children, Journal of Laws of 2020, item 14,100. The above-mentioned constitutional norm is further developed by numerous statutory provisions that shape appropriate branch relations (with the Family and Guardianship Code (F and GC) at the forefront), allow for the application of appropriate administrative mechanisms in the event of a threat to the child’s welfare (e.g., on supporting the family and the system of foster care, Journal of Laws of 2022, item 447). In its preamble, it was emphasized that this act was adopted “for the benefit of children who need special protection and help from adults, the family environment, an atmosphere of happiness, love and understanding, for the sake of their harmonious development and future independence in life, to ensure the protection of their rights and freedom, and are strictly devoted to preventing and combating the so-called domestic violence.” (Act of 29 July 2005 on counteracting domestic violence, Journal of Laws of 2021, item 1249), and it also provides penal sanctions for behaviors that endanger or violate the interests of minors (mainly the Penal Code (PC).

The limited framework of the study does not allow for an in-depth analysis of all these regulations. Therefore, further considerations will be directed only to selected family and criminal law aspects, as well as the obligation to denounce in the context of medical secrecy.

### 5.2. Family and Legal Aspect

Pursuant to Art. 72 sec. 1 of the Polish Constitution, is the so-called the good of the family. The Constitutional Tribunal, explaining this term, pointed out that this ‘good’ is characterized by “durability, which forms the basis for the sense of security of all her [family] members, especially the weakest-children, sick or disabled people. The good of the family is created by strong and lasting bonds-positive emotional relations connecting its members, favoring their personal development and feeling happy because of the closeness with other family members” [70]. The family is therefore subject to special protection and is a subject of care by the state. This constitutional regulation is supplemented by the provisions of family law, which define subsidiary relationships, in particular between parents and children. Therefore, Art. 95 § 1 of the Civil Code parental authority includes, in particular, the obligation and right of parents to take care of the child’s person and property and to raise the child, respecting their dignity and rights. In turn, art. 96 § 1 of the Civil Code states that parents are obliged to take care of their child’s physical and spiritual development and to prepare them properly to work for the good of society according to their talents. The literature explains that this concern includes providing the minor with an appropriate material environment and protection against dangers. In particular, parents should take care of the child’s health, provide them with care and treatment, and living conditions and entertainment appropriate to their age. It is also indicated that protection against threats concerns both dangers coming from third parties, e.g., against a harmful environment, gangs, sects, and from the forces of nature, e.g., natural disasters [71]. Therefore, if parents are to protect their child against such events, they cannot be they cannot be the source of such harm. Thus, they cannot undertake such activities that would jeopardize the child’s welfare, in particular, they may not use violence against the child. It cannot be used instrumentally, as a tool to enforce obedience, or as an educational measure. This issue is discussed in Art. 961 F&GC, which prohibits persons exercising parental authority and custody or custody of minors from using corporal punishment. The jurisprudence makes it clear that “the systematic abuse of a child for the sole purpose of ensuring the possibility of stress-free” custody “of a child is a motive that does not find any rational justification and thus is trivial. Willful abuse of a small child, deliberately causing serious damage to his health, and finally causing his death inadvertently, are the most serious crimes in the society’s perception” [72]. It is emphasized that “parents do not have the right to use violence against children in the event of educational problems”, although “not every educational failure of this type should be classified as abuse within the meaning of Art. 207 § 1 of the CC“ [73]. Criminal law intervenes only in cases of serious infringement/exposure of legal goods including a minor (the so-called ultimate measure function-ultima ratio). On the basis of family law, however, the legislation also provided for certain intervention instruments. The most serious of these is the deprivation of parental authority. Pursuant to Art. 111 § 1 of the Civil Code, the court may order this measure, inter alia, when the parents abuse parental authority or grossly neglect their obligations towards the child. The literature explains that the first condition is met if the parents use corporal punishment against their children, use them for sexual immorality, including prostitution, or commit a crime to the detriment of a minor, particularly physical or mental abuse, and drinking. On the other hand, neglect may include abandoning a minor and leaving a young child in life-threatening conditions (e.g., on a public road, on railroad tracks) [74]. Similarly, these conditions are considered in the judicature, where it is considered that they are met in the case of alcohol abuse or a criminal activity against a child [75]. Moreover, the case-law clarifies that the application of the measure in question can only take place where ‘the neglect of the obligations towards the child … can be assessed as gross’. Serious negligence or negligence of a minor, which takes on the characteristics of incorrectness and persistence, must be recognized. The consequence of such a court decision is the total loss of parental authority over the child (which, however, does not mean an automatic prohibition of contact with the child). If the above-mentioned conditions do not occur, but there are less serious irregularities in the process of directing and caring for the child, the guardianship court may apply other instruments, provided for in Art. 109 F&GC. The condition for making use of this provision establishes that the child’s welfare is at risk. Such an approach to the provision indicates its preventive application, as it is sufficient to create a threat to the child’s interests, and it is not necessary a means to violate them. A minori ad maius, the court may undoubtedly use this regulation also when there has already been a violation against the interest of the child, e.g., acts of violence against a minor, and especially when there is a probability of their recurrence. Then, the court may issue an appropriate order. An exemplary enumeration of such activities is included in Art. 109 § 2 of F&GC. In the context of violence against a minor, it is possible, among other actions, to oblige the parents and the minor to specific actions, i.e., to work with a family assistant, carry out other forms of work with the family, refer a minor to a day support facility or refer parents to an institution or a specialist dealing with family therapy, counseling or providing other appropriate help to the family. Moreover, the court, perceiving the need to change the child’s environment, may decide to place the child in a foster family, family orphanage or institutional foster care.

Proceedings in the above-mentioned cases are initiated ex officio. However, everyone who learns about a threat to the child’s welfare is obliged to notify the guardianship court about it (see the last fragment of the study for more details), which will allow the court to become aware of the need for appropriate intervention.

The Polish Penal Code does not define the concept of violence, but in many provisions it treats violent behavior as a means to achieve its goal by the perpetrator (the crime of coercion—Art. 191 of the Penal Code) or to inflict harm on the victim. The behaviors listed in the first part of this study may therefore correspond to the descriptions of various prohibited acts. For example, they can be classified as crimes against health (e.g., causing light or moderate bodily—Art. 157 of the Criminal Code, possibly serious damage to health—Art. 156 of the Criminal Code), against sexual freedom (in particular rape of a minor under 15 years of age—Art. 197 § 3 of the Criminal Code and the so-called sexual abuse of a minor—Art. 200 of the Criminal Code), and against honor and bodily inviolability (insult—Art. 216 of the Criminal Code and violation of bodily inviolability—Art. 217 of the Criminal Code). It seems, however, that the crime of abuse described in Art. 207 PC.

In dictionary terms, the verb “to bully” is “to make someone suffer (physical, moral), torment someone” [76]. This is perceived in a similar way in jurisprudence. For example, one can cite the resolution of the Supreme Court (the entire chamber of the Supreme Court—the Criminal Chamber) of 9 June 1976 [77], in which it was explained that the “statutory term bullying means an act or omission consisting of deliberately inflicting physical pain or acute moral suffering, repeated or one-time, but intense and extended over time”. Thus, the judicature admits that bullying may consist of a multiplicity of behaviors as well as a single behavior, but one that is characterized by a sufficiently high intensity of inflicted suffering [78]. It is characteristic of bullying that “the perpetrator has an advantage over the aggrieved party which he or she cannot oppose or can do so to a small extent” [79].

This property makes it possible to distinguish bullying from other criminal behavior, e.g., violation of bodily integrity and insults. The literature considers the criterion for assessing the occurrence of bullying. It is indicated that the interpretation of this characteristic may be based on the victim’s subjective point of view (her feelings) and on the basis of an objective criterion (assessment from the perspective of third parties) [80]. However, these sensations can be very different and depend on the victim’s threshold of sensitivity. Criminal law protects legal goods regardless of the subjective view of the aggrieved party, as it is aimed at assessing the social harmfulness of the act. Therefore, the doctrine proposes that the overall social criterion should be used to assess the occurrence of bullying, focused on objectified assessments [81].

This issue seems important in the context of the topic of this study. Persons experiencing violence, due to their mental state, age, as well as social and cultural conditions in the environment from which they come, may misread the perpetrator’s behavior and fail to perceive forms of violence corresponding to the concept of abuse (especially when the process occurs over a long time and the victim becomes “immune” to such behavior). For the existence of this crime, it does not matter whether the victim objected to such behavior and undertook any defensive measures [82]. This thesis is of great importance in the case of juvenile victims. Due to their age and power disproportion, they cannot oppose the perpetrator, and when fearing an escalation of violent actions, they do not attempt a defensive action.

Pursuant to § 1 of the discussed article, bullying may be physical or psychological. An alternative approach to causative behavior leads to the conclusion that in order to bear responsibility for bullying, one of these forms of violence is sufficient, although the complex of causative activities may consist of both actions aimed at causing physical as well as moral (mental) ailments. These concepts are evaluable and need to be analyzed in concerto. However, the doctrine exemplifies what physical and psychological abuse is. With regard to the first form, it is indicated that the causative behavior may be manifested by inflicting painful physical suffering, for example, beating, tying the legs, or exposing to the cold [83]. In this approach, violence may also take the form of omission, i.e., failure to take actions that a person is obliged to do, for example, not providing food, clothes, or adequate housing conditions for minors [84]. It is more difficult to identify the occurrence of psychological abuse. The literature explains that it consists in causing “mental discomfort in the victim”, arousing “in her a sense of threat, anxiety, fear for her own fate and property”. It can take the form of scaring, insulting, using invectives, making life unpleasant, and humiliation [85]. With regard to minors, it is explained that psychological abuse may consist of mental neglect, and deliberate failure to meet their physical, emotional, intellectual or material needs [86]. The violence can also be indirect, directed at animals or children’s things. The behavior of the perpetrator is then aimed at awakening unpleasant sensations in the victim in connection with the loss of a beloved animal or favorite object. This issue was pointed out by the Supreme Court, which examined the case of killing a guinea pig and a canary belonging to the perpetrator’s child with an air rifle. The court stated that “the accused consciously and deliberately killed these animals and it was an element of mental abuse of his family members. His action was also dictated by the desire to hurt family members, to demonstrate his strength, a sense of impunity and submission to his will. The violence used in this case was directed to the environment of the aggrieved persons, that is animals, and was aimed at influencing their awareness and will” [87]. The court therefore concluded that it was a symptom of mental abuse.

The act in question is of the formal nature (unsuccessful). It is therefore not necessary for the aggrieved party to suffer any health detriments in order to perform it. Bullying behavior itself is penalized. The legislation, however, envisages a type of this act qualified by succession. The punishment is therefore more severe when the abuse results in the victim taking their life (Article 207 § 3 of the Criminal Code).

In addition to penalties against the perpetrator of this crime, it is possible to introduce criminal measures, in particular the prohibition of contacting specific persons, approaching certain persons and an order to periodically leave the premises shared with the aggrieved party (Article 41a of the Criminal Code). Moreover, a court sentencing for this act may apply Art. 43c PC. Pursuant to it, the court deciding that it is necessary to deprive or limit parental or guardianship rights in the event of committing a crime to the detriment of a minor or in cooperation with him, notifies the competent family court. Such notification will launch the instruments provided for in the Family and Guardianship Code, discussed in the previous point.

The offense in question is prosecuted under the terms of public prosecution. In order to initiate the proceedings, it is sufficient for any person, including a medical employee, to submit a notification to the authorities appointed to prosecute crimes (e.g., at the police). However, in the case of this group of people, the matter becomes more complicated due to the binding professional (medical) secrecy. The relationship between the obligation to denounce and medical discretion will therefore be discussed in the Section 5.3.

### 5.3. Obligation to Denounce in the Case of Violence against Minors and Medical Secrecy

Historically, it has been emphasized that the fundamental duty of a doctor (and later also of other people involved in providing medical aid) is to keep secrets. This obligation has been articulated, among others, in in the Oath of Hippocrates, “Whatever during treatment or besides treatment in people’s lives I see or hear that cannot be revealed, I will keep silent about it, having it for a sacred secret.” Nowadays, there is a multi-faceted justification of this duty. The duty of discretion has a utilitarian dimension (the conventionalist concept) and shapes the appropriate relationship between the patient and his physician (the fidelity theory in mutual relations) based on respect for the patient’s dignity and subjective treatment. In Polish law, the foundations of the obligation to keep medical secrets can be found as constitutionally protected privacy. This obligation is therefore derived from Art. 47 of the Polish Constitution, which states that everyone has the right to legal protection of their private life and to make decisions about their personal life. In this area there are, among other information, data on the state of health and medical procedures performed [88]. The constitutional basis of medical confidentiality results in the application of the principle of proportionality, as laid down in Art. 31 sec. 3 of the Polish Constitution. It can facilitate the limitation of civil rights and freedoms (including those relating to the protection of privacy) only by statutes and only when it is necessary to protect other precious values (the limitation clauses), e.g., the health of other people. It follows that any exceptions (dispensations) to medical discretion must be stipulated by law and must not be interpreted broadly.

On the basis of medical law, the issue of medical secrecy is treated both from the perspective of patients’ rights (this issue is covered by Articles 13 and 14 of the Act of 6 November 2008 on patient rights and the Patient Rights Ombudsman (PRO), Journal of Laws of 2020, item 849, as amended), as well as corporate acts constituting an order of discretion applicable to representatives of a given medical profession (e.g., Article 40 of the Act of 5 December 1996 on the professions of a physician and dentist (APP&D)“, Journal of Laws of 2021, item 790, as amended). These provisions define the subjective and objective scope of secrecy and outline instances in which it can be suspended. For the record, it is worth explaining that in the light of these regulations, the material scope of a secret is defined very broadly. It covers not only strictly medical data, but also other information that a medical worker obtains while performing professional duties (e.g., about the patient’s family status, financial situation, profession, hobbies, etc.) [89]. Therefore, the catalog of confidential data may also contain information about the violence experienced by the patient and its physical and mental consequences. A question then arises about the necessity/possibility of notifying the relevant authorities, which will take actions aimed at the protection of the victim of violence (especially minors), such as punishing the perpetrator. In criminal law, this issue is considered in the context of the obligation to denounce, i.e., to notify about a disclosed prohibited act. There are two types of this duty, i.e., the legal and social obligation. The implementation of the former is secured by sanctions, in particular criminal sanctions. It is therefore assumed that it takes precedence over the obligation of discretion. A medical worker must therefore provide data on the disclosed act to the authorities established to prosecute crimes, regardless of medical secrecy. On the other hand, the social duty has only a moral and civic dimension, so its performance is not secured by sanctions. Hence, it is considered to be inferior to the obligation of medical secrecy. Take for example, a person providing health services who, despite the confidentiality that is binding, would like to notify the police. In order to justify the breach of discretion, they must therefore refer to an additional dispensation resulting from medical law regulations, e.g., the patient’s consent. The mere reference to the fulfillment of the social obligation to denounce is not sufficient to legalize the violation of discretion. It is therefore crucial to determine when a medical worker has a legal obligation and whether it concerns manifestations of violence against minors.

The most important basis of the legal obligation is Art. 240 § 1 of the Polish Criminal Code, which enumerates (i.e., as a closed catalog) the generic types of prohibited acts subject to denunciation. Referring to this enumeration as the subject of the study, it can be indicated that this obligation will apply to serious damage to health (Art. 156 of the Criminal Code). It involves, among other things, causing “other severe disability”, which is understood as a serious limitation of the functions of a vital organ such as heart, lungs, bones of the skull; damage to the limb, causing its paresis, causing amblyopia through damage to the eyeball, etc. [90] or “a truly life-threatening disease”. It may arise as a result of a one-off behavior of the perpetrator, e.g., a strong blow. Examples of injuries that may cause the disease in question are: contusion of the brain, stab wounds in the abdomen, cranial injuries in the form of a fracture of the occipital bone and other serious disorders of basic functions of the systems, e.g., respiratory or circulatory. This group also includes concussion associated with long-term loss of consciousness, injury to large arteries, and perforation of the digestive system walls [91]. Such an injury does not have to cause a long-term disease state or an incurable disease. Undoubtedly, such health effects may arise as a result of serious domestic violence (e.g., by hitting the head with a heavy object, stabbing with a sharp instrument, hitting the head with a glass bottle or hammer, etc.) [92]. Moreover, some offenses against sexual freedom are legally denounced. This group includes the qualified type of rape (e.g., against a minor under the age of 15-Art. 197 § 3 point 2 of the Criminal Code) and the sexual abuse of a minor under the age of 15 (Article 200 of the Criminal Code). In the context of violence, it is worth noting that this act is committed not only when the perpetrator coerces sexual intercourse of such a minor, but also when other sexual activity occurs. The Supreme Court explained that “such behavior, not falling under the concept of” sexual intercourse “is related to the broadly understood sexual life of a human being, consisting in the perpetrator’s physical contact with the aggrieved or at least on the bodily and sexual involvement of the victim” [93]. For example, it may consist in kissing, caressing, touching the genitals (even through underwear or clothing), breasts, exposing the body in a sexual context, inserting various objects into the vagina or anus, etc. [94]. For the existence of this crime, it is not important whether the victim has sustained any bodily injuries or other consequences for physical or mental development [95].

It is worth emphasizing that in the catalog of acts included in Art. 240 § 1 of the Criminal Code, the legislation did not indicate any abuse. The obligation to notify about this offense is therefore only of a social nature. As indicated, it gives way to the obligation of keeping medical secrecy. A person providing health services, noticing the symptoms of abuse and wishing to notify the authorities appointed to prosecute crimes, must additionally refer to one of the dispensations provided for in medical and legal regulations. Therefore, it is possible to rely on the consent of the patient (adult and non-incapacitated), or in the case of a minor—upon the consent of his legal representative (usually parents). The provisions, however, provide for another exception that may apply in the situation under discussion—namely, if keeping the secrecy may pose a threat to the life or health of the patient or other persons (e.g., Art. 14 (2) (2) of the PPA and Art. 3 APP&D). The doctrine explains that a medical worker may take advantage of the dispensation in question only when the threat is real and serious and does not result from the dynamics of the disease (it must therefore be “external”) [96]. In the context of violence against minors, the reasoning would then be as follows: failure to disclose such violence and failure to inform the competent authorities will result in the victim continuing to be subjected to it, which in turn may endanger at least their health. However, the information obtained about the patient’s health should justify the conviction that he may actually be at such a risk (e.g., a pediatrician recognizes an injury to a child resulting from beating by a parent). However, the matter becomes more complicated in the case of persons performing their tasks under the Act of 19 August 1994 on the protection of mental health (Journal of Laws of 2020, item 685, as amended, The Mental Health Protection Act (MHPA)). Art. 50-52 regulate the rules of behavior of the so-called a psychiatric secrecy that is more strict than medical discretion. Pursuant to Art. 50 sec. 1 is related to all persons performing activities under this Act. Thus, the doctrine explains that the obligation of confidentiality covers not only medical workers, but also administrative staff, psychologists and representatives of law enforcement and justice authorities involved in the activities provided for in the Act (e.g., visiting judge) [97]. In general, it can be indicated that this regulation is addressed to all people employed in psychiatric health care, as well as to other entities, such as those participating in “didactic classes, in scientific and research work” and performing “tasks related to social assistance for people with mental disorders” [98]. In the subject matter, this mystery covers “everything about (these persons-add. aut.) will announce the message in connection with the performance of these activities.” It is therefore a very broad outline of this duty. Due to the specific nature of psychiatric secrecy (its preservation is particularly important for the proper development of the relationship between the psychiatric patient and the person providing for him/her, based on a strong relationship of trust), the doctrine indicates that this type of discretion takes precedence even over the legal obligation of denunciation, referred to in art. 240 §1 of the Criminal Code [99]. Therefore, the question arises as to whether such a person does not have to notify authorities about the disclosed prohibited act, and whether he or she can do so at all. For this to be permissible and a person not to be held liable for breach of discretion (even criminal—Art. 266 § 1 of the Criminal Code), they would have to justify themselves by means of some dispensation. The list of psychiatric secrecy is included in Art. 50 sec. 2 of the Public Procurement Law. However, neither the consent of the patient nor an exception are required when keeping a secret may endanger the patient’s life or health. It seems that in the former case such a gap is easy to fill. If it is considered that the patient is the administrator of the data concerning him, he can freely decide whether and to whom it will be disclosed. The patient’s consent is therefore a fairly obvious ground justifying the breach of discretion. Provided, however, that the patient has the appropriate formal (adult) and factual competence (their mental state will allow them to make a declaration effectively, with discernment and awareness of this act). It is more difficult to explain the possibility of invoking the second dispensation. A question arises whether a medical worker, having learned about such a danger (caused by violence against a patient), is released from secrecy and should notify the appropriate authorities. Various positions on this subject are presented in the literature. One can meet the opinion that, due to the specificity of psychiatric secrecy, persons employed in psychiatric care, provided that they obtain information under the conditions of Art. 50 sec. 1 of the Public Procurement Law, are bound by a secret which does not relieve the risk to the life or health of the patient. This act is a specific regulation in relation to corporate regulations [97] (pp. 241–243). However, the opposite seems to prevail. It is argued that one should refer to the systemic and teleological interpretation. It is also indicated that adopting the primacy of the Mental Health Protection Act, and thus excluding the possibility of revoking a psychiatric secret due to the threat to the patient’s life or health, would have serious consequences. It would require discretion even in cases of serious danger. The norms of the Mental Health Protection Act only supplement the general regulation resulting from other acts. It is therefore explained that “a psychiatrist has the same obligations to disclose information protected by medical confidentiality as any other doctor [provided for in Art. 40 sec. 2 APP&D-perm. aut.], and also additional ones (e.g., the necessity to provide this information to the state security services provided for in Article 50 (2) (4) of the Protection Regulation)” [100].

The presented argument shows that the relations between the Mental Health Protection Act and other medical and legal regulations that establish the principles of maintaining medical secrecy are quite unclear. If the former act were to be considered a lex specialis, it would, in fact, displace the application of other general regulations. Thus, the analyzed dispensation could not be used. However, it is possible to refer to another rule of interpretation, namely the lex posterior derogat legi priori, whereby the newer act supersedes the older act. It is worth noting that both the Act on the professions of a physician and dentist, and the Act on patient rights and the Patient Rights Ombudsman, are newer than the Act on the protection of mental health. According to this principle, they would therefore take precedence over. Moreover, adopting the primacy of psychiatric secrecy would lead to systemic inconsistencies. The same medical worker, depending on the type of disease and actions taken in connection with it, would be subject to different legal regimes. Thus, the most recent literature allows for the possibility of waiving discretion in the discussed circumstances, indicating that the possible liability for revealing a secret may then be excluded based on the construction of a state of higher necessity [101].

However, this does not change the fact that the relationship between the regulations in question is not clarified. Therefore, the legislator’s interference and an unequivocal solution to these problems are desirable.

Apart from the provisions referred to above, obliging the authorities appointed to prosecute crimes to be notified, it should be noted that other regulations also constitute a social obligation in this respect. First of all, it is necessary to indicate Art. 304 § 1 of the Code of Criminal Procedure, according to which everyone, having learned about the commission of an offense prosecuted ex officio, has a social obligation to notify the prosecutor or the police about it. A similar structure is contained in Art. 12 sec. 1 of the Act of 29 July 2005 on Counteracting Domestic Violence. It obliges all persons who, in connection with the performance of their official or professional duties, suspect that an ex officio prosecuted crime involving domestic violence has been committed shall immediately notify the police or the prosecutor of this fact. Therefore, the obliged entities also include medical workers. However, it should be emphasized once again that these regulations only constitute a social obligation to denounce. Therefore, the disclosure of discrete data requires reference to one of the dispensations provided for in the medical and legal regulations. These exceptions will also apply in the event of the need to notify the guardianship court. Such an obligation results from Art. 572 of the Code of Civil Procedure. Pursuant to § 1 thereof, anyone who knows of the event justifying the initiation of proceedings ex officio is obliged to notify the guardianship court about it. This obligation is addressed, among others, to institutions caring for children or mentally ill people. On the other hand, the wording of § 2 may conclude that the enumeration of the obliged entities is only exemplary (the provision uses the formula “primarily”). Hence, the literature indicates that this obligation also rests on other entities, not indicated in this provision, and therefore also on natural persons [102]. This solution is also applicable to medical workers. They are required to provide such notification in cases where the guardianship court may institute proceedings ex officio. This category includes some family matters and care matters, incl. deprivation of parental authority (Article 111 of the Civil Code). In turn, it can take place, e.g., if the parents abuse parental authority or grossly neglect their obligations towards the child. In turn, as already explained in the previous fragment of the study, such abuse may correspond to the manifestations of domestic violence and other prohibited acts that harm the health or bodily integrity of the child and its sexual freedom. Therefore, if a medical worker discloses such circumstances, he or she is obliged to notify the guardianship court about them. Although the mentioned provision does not specify the date by which such a notification should take place, it should be assumed that due to the urgent need to initiate proceedings in such cases, the message should be delivered as soon as possible. This will allow the guardianship court to take appropriate measures to protect the child’s welfare. Considering, however, that there is only a social obligation, it is necessary to additionally refer to some exceptions to medical secrecy.

The limited framework of the study did not allow for a broader analysis of foreign legislation, although the acts of presented international law lead to the conclusion that much attention is devoted to the issue of protecting children against violence. Not only the bodily integrity, but also the dignity of the minor is protected.

These provisions are in the form of certain declarations, although they oblige the states party to them to implement appropriate mechanisms and legal solutions in their national legislature. Therefore, it is of key importance that the signatories of the established international standards perform these obligations.

By evaluating Polish regulations, it can be noted that they are multifaceted. Indeed, the protection of the child takes place both in the family-legal sphere and on the basis of criminal law. The initiation of proceedings in the latter sphere is served by the legal obligation of denunciation. However, these norms are not perfect; in particular, this duty is not extended to the most relevant—from the point of view of the issue at hand—crime of abuse. Therefore, one can postulate amendments to Article 240 of the Criminal Code to add this offense to the catalog of acts subject to legal notification. The norms on the denunciation obligation are also not coherent with the provisions governing psychiatric secrecy. Indeed, it is not clear whether, in a case in which the preservation of medical (psychiatric) discretion could result in danger to the health or life of the patient or others, it is possible to violate psychiatric secrecy by notifying the authorities established for criminal prosecution (for example, if the victim of such an act is a child who is found to have a mental disorder as a result of the act). For the sake of systemic consistency and because of the need to clearly delineate the limits of the denunciation obligation incumbent on persons providing psychiatric assistance, it is desirable that the legislator regulate this matter unambiguously. This will remove the dilemmas of those working in psychiatric care regarding the proper handling of such cases, i.e., whether psychiatric confidentiality or the duty to denounce takes precedence. De lege ferenda, therefore, it would be appropriate to postulate appropriate amendments to the Law on Mental Health Care or to the Law on Patients’ Rights and Patients’ Ombudsman in the section on medical confidentiality.

However, it seems that the introduction of relevant regulations alone will not be sufficient. After all, for their efficient and effective application, it is necessary that they be made aware to the addressees of these norms, especially members of the medical staff who may uncover instances of violence against children. In addition, it is necessary to internalize these regulations so as to understand the need and desirability of the protective measures described in these regulations. Thus, it is desirable, firstly, to inform as widely as possible about the legal regulations in this area, and secondly, to take educational measures so as to form appropriate social attitudes when manifestations of violence against children are revealed. Undoubtedly, the role of medical professionals in this area is invaluable and momentous. Therefore, it is worthwhile for these people not to hesitate and actively take steps to protect minors from violence and its physical and psychological consequences.

## 6. Summary

There is no country or community unaffected by violence—it permeates the media, and is present at home, in schools, work and institutions. It is a universal problem that threatens the life, health and happiness of all of us. Culture plays a key role in this respect, as it sets the boundaries of acceptable behavior, determines when a behavior is considered violence, and indicates how to respond to it. Examples include differences around the world in terms of attitudes towards punishing children, assessing sexual violence against women (in some countries raped women are not protected by law and may be killed by their families for honorary reasons), forced marriage, etc. Every year, more than 1.6 million people worldwide lose their lives due to violence. Many more have psychological, sexual and psychological problems for the same reason [103]. Long-term consequences of the use of violence against children are already observed on the biological-level changes in the structure of the brain. Based on the analysis of brain scans of people who experienced or did not experience violence in childhood, it was found that there is a significant loss of gray matter cells in the brains of people affected by violence, especially in the area responsible for memory and cognitive control. This explains why children experiencing violence have learning difficulties or become more aggressive or, conversely, are very submissive, especially in stressful situations [104]. Victims of violence are at an increased risk of developing both mental disorders, e.g., depression and anxiety disorders, eating disorders as well as violent and aggressive behavior, alcohol or other psychoactive substance abuse. It has been shown that the impact of violence on depression among children and adolescents is sustained. Accordingly, evidence suggests that elevated depressive symptoms and episodes of depression may persist for up to two years after experiencing cases of violence [6]. The costs associated with the consequences of violence weigh on health institutions. In view of the above, it is so important that due to the destructive consequences of such behavior, international and national law devote attention to the protection of children rights to apply appropriate regulations in this regard.

## Data Availability

Not applicable.
